# Combined use of preoperative ^18^F FDG-PET imaging and intraoperative gamma probe detection for accurate assessment of tumor recurrence in patients with colorectal cancer

**DOI:** 10.1186/1477-7819-5-80

**Published:** 2007-07-16

**Authors:** Ismet Sarikaya, Stephen P Povoski, Osama H Al-Saif, Ergun Kocak, Mark Bloomston, Steven Marsh, Zongjian Cao, Douglas A Murrey, Jun Zhang, Nathan C Hall, Michael V Knopp, Edward W Martin

**Affiliations:** 1 Division of Nuclear Medicine, Section of PET, Department of Radiology, The Ohio State University, Columbus, OH 43210, USA; 2 Division of Surgical Oncology, Department of Surgery, Arthur G. James Cancer Hospital and Richard J. Solove Research Institute and Comprehensive Cancer Center, The Ohio State University, Columbus, OH 43210, USA; 3 Department of Radiology, Medical College of Georgia, Augusta, GA 30912, USA

## Abstract

**Background:**

The purpose of this study was to combine intraoperative gamma probe (GP) detection with preoperative fluorine 18-fluoro-2-deoxy-glucose positron emission tomography (^18^F FDG-PET) imaging in order to improve detection of tumor recurrence in colorectal cancer (CRC) patients.

**Methods:**

Twenty-one patients (12 females, 9 males) with a mean age of 54 years (range 31–78) were enrolled. Patients were suspected to have recurrent CRC by elevated CEA (n = 11), suspicious CT findings (n = 1), and clinically suspicious findings (n = 9). Preoperative FDG-PET scan and intraoperative GP study were performed in all patients. Mean time interval between preoperative FDG-PET scan and surgery was 16 days (range 1–41 days) in 19 patients. For intraoperative GP studies, 19 patients were injected with a dose of 10–15 mCi ^18^F FDG at approximately 30 minutes before the planned surgery time. In two patients, the intraoperative GP study was performed immediately after preoperative FDG-PET scan.

**Results:**

Preoperative FDG-PET and intraoperative GP detected 48 and 45 lesions, respectively. A total of 50 presumed site of recurrent disease from 20 patients were resected. Thirty-seven of 50 presumed sites of recurrent disease were histological-proven tumor positive and 13 of 50 presumed sites of recurrent disease were histological-proven tumor negative. When correlated with final histopathology, the number of true positive lesions and false positive lesions by preoperative FDG-PET and intraoperative GP were 31/9 and 35/8, respectively. Both preoperative FDG-PET and intraoperative GP were true positive in 29 lesions. Intraoperative GP detected additional small lesions in the omentum and pelvis which were not seen on preoperative FDG-PET scan. FDG-PET scan demonstrated additional liver metastases which were not detected by intraoperative GP. Preoperative FDG-PET detected distant metastasis in the lung in one patient.  The estimated radiation dose received by a surgeon during a single 18F FDG GP surgery was below the occupational limit.

**Conclusion:**

The combined use of preoperative FDG-PET and intraoperative GP is potentially helpful to the surgeon as a roadmap for accurately locating and determining the extent of tumor recurrence in patients with CRC. While intraoperative GP appears to be more sensitive in detecting the extent of abdominal and pelvic recurrence, preoperative FDG-PET appears to be more sensitive in detecting liver metastases. FDG-PET is also a valuable method in detecting distant metastases.

## Background

Colorectal cancer (CRC) remains the third leading cause of new cancer cases and the third leading cause of cancer deaths for both men and women in the United States [1]. Up to 50% of all CRC recurrences develop within two years of the time of the initial surgery intervention [2-4]. In this regard, it is estimated that up to 50% of these local recurrences may be suitable for attempted surgical extirpation [2-10]. Therefore, the accurate detection and localization of all sites of tumor recurrence plays a critical role in helping physicians to select the most appropriate therapies to ultimately impact upon long-term patient outcomes

The use of fluorine 18-fluoro-2-deoxy-glucose positron emission tomography (^18^F FDG-PET) scan imaging as part of the diagnostic work-up of patients with rising carcinoembryonic antigen (CEA) is well-established [11-14]. FDG-PET has been shown to be superior to CT and MRI for detecting local recurrences, as well as for detecting metastatic disease to the liver and to distant sites [15-21]. In a recent meta-analysis, FDG-PET was determined to have an overall sensitivity of 97% and overall specificity of 76% for detecting recurrent CRC [22]. Nevertheless, current generation FDG-PET systems still have the limitation of not detecting small-volume disease [23].

The concept of radioguided surgery employs the use of a preoperative injection of a radiolabeled tumor imaging agent and intraoperative detection of tumor via a hand-held gamma probe (GP). Intraoperative GP studies with radiolabeled monoclonal antibodies have previously demonstrated that this method provides immediate intraoperative staging, defines the extent of nodal involvement, delineates resections margins, and localizes sites of occult disease in patients with primary or recurrent CRC [24-27]. Furthermore, ^18^F FDG detection using the intraoperative GP has been shown to be feasible and correlates well with preoperative FDG-PET imaging for detecting sites of disease in CRC patients [28-30].

In the current study, we have set out to combine intraoperative GP detection with preoperative FDG-PET imaging in order to attempt to improve intraoperative tumor detection in patients with findings that are highly suspicious for recurrent CRC.

## Methods

### Patients

This study protocol was approved by the Ohio State University (OSU) Comprehensive Cancer Center Scientific Review Committee and by the Cancer Institutional Review Board of the Arthur G. James Cancer Hospital and Richard J. Solove Research Institute and Comprehensive Cancer Center of OSU.

The intraoperative GP study was performed in 21 patients with presumed recurrent CRC, based on a positive preoperative FDG-PET scan. Likewise, 11 of these patients had an elevated preoperative CEA level, one had a suspicious CT scan, and nine had clinically suspicious findings. The characteristics of the patients are summarized in Table 1.

**Table 1:** Patient Characteristics

**Characteristics**	**Patients**
Mean age [range (year)]	54 (31–78)
Gender	
Males	9
Females	12
Mean time interval from initial treatment to PET scan [range (months)]	42 (3–102)
Mean time interval from PET scan to Surgery/GP study [range (days)]	16(1–41)(19 patients)

### FDG-PET imaging

FDG-PET imaging was performed as either combined PET/CT studies on a Siemens Biograph 16 PET/CT camera (CTI, Inc, Knoxville, TN) or as PET only studies on a HR plus Siemens CTI PET camera (CTI, Inc, Knoxville, TN). Combined PET/CT studies were performed in 18 patients and PET only studies were performed in 3 patients. The patients fasted approximately 6 hours prior to intravenous injection of 370 to 555 MBq (10 to 15 mCi) ^18^F FDG. Blood glucose levels were checked prior to the injection of ^18^F FDG. Studies were performed only when blood glucose levels did not exceed 150 mg/dL. The imaging sequence was started approximately 60 minutes following the intravenous injection of ^18^F FDG. For the combined PET/CT studies, first a scout view was obtained with 30 mAs and 130 kVp followed by a spiral CT scan with 130 mAs, 130 kVp, 5 mm scan width, and 12 mm feed per rotation without any specific breath-holding instructions. No IV or oral contrast was given to the patients for the CT portion of the study. Imaging area was from the skull base to the proximal femoral region. FDG-PET scanning was performed immediately after acquisition of the CT images without changing the patient position with 2 to 4 minutes per bed acquisition time. FDG-PET images were corrected for attenuation on the basis of the CT data, and iterative reconstruction algorithms were used for reconstruction. For the PET only studies, image acquisition time of 10 minutes per bed by using 40% transmission and 60% emission protocol was used. FDG-PET images were corrected for attenuation on the basis of the transmission image data, and iterative reconstruction algorithms were used for reconstruction. All FDG-PET images were evaluated by two board-certified nuclear medicine physicians. Quantification of the tumor metabolic activity was obtained using the Standardized Uptake Value (SUV) normalized to body weight.

### Intraoperative GP studies

In 19 patients, the intraoperative GP studies were done a time that was chronologically remote from the time of the preoperative FDG-PET scan. In these 19 patients, the mean time interval between the preoperative FDG-PET scan and date of surgery for intraoperative GP was 16 days (range 1 to 41 days). These 19 patients were intravenously injected with 370 to 555 MBq (10 to 15 mCi) of ^18^F FDG at approximately 30 minutes before the planned surgery time. However, in two patients, the intraoperative GP study was performed within 30 to 60 minutes after the completion of the preoperative FDG-PET scan, and no second dose of ^18^F FDG was administered.

The intraoperative GP device utilized was the Neoprobe neo2000 unit (Neoprobe Corporation, Dublin, Ohio). Intraoperatively, the GP device was used to systematically obtain GP counts for all suspicious areas within the entire operative field of interest. All examined areas with GP counts determined to be greater than 3 standard deviations above the background counts were considered abnormal tissue. Lesion Count/Background Count (LC/BC) ratios were calculated for lesions detected by the GP.

### Statistical analyses

Mean ± standard deviation (SD) of maximum-pixel SUV (SUV_max_) and mean ± SD of LC/BC ratios of the lesions were calculated. The significance of these values between false positive and true positive lesions was compared by t-test. A P-value of less than or equal to 0.05 was considered statistically significant.

### Calculation of estimated radiation dose to the surgeon during GP surgery

To estimate the ^18^F radiation dose to a surgeon, we measured radiation dose rates for several patients using a Victoreen 450 P ionization chamber calibrated. The measurements were taken at three distances, 0.152 m (6 inches), 0.305 m (12 inches) and 0.914 m (36 inches) from patient's abdomen either on the right or left side. The average elapsed time from the time of measurement to the time of the ^18^F FDG injection was approximately 107 minutes. The measurements resulted in an average dose rate of 163.7 μGy/hr at 0.152 m from the patient, 105.0 μGy/hr at 0.305 m, and 18.5 μGy/hr at 0.914 m.

Using the measured dose rates above, we calculated the cumulative radiation dose to the surgeon at the three distances (0.152 m, 0.305 m and 0.914 m) for 1, 2, 3, 4, and 5 hour duration of operation. Since the surgery started either approximately 30 minutes after or approximately 3 hours after the ^18^F FDG injection, we carried out calculation for both scenarios. Since the duration of operation generally varied from one to five hours, we calculated a cumulated radiation dose for five time intervals of 1, 2, 3, 4, and 5 hours.

In the calculation, we only took ^18^F physical decay (T_1/2 _= 110 m) into account and ignored the change in FDG biodistribution that may have occurred during the elapsed time. We also made a theoretical assumption that the patient had no urine voiding during this time period, so that the estimate that was calculated would represent the upper limit for radiation dose to the surgeon.

We first calculated the dose rate D(t) at different time t, such as t = 30, 90, 150, 210, 270, and 330 minutes, using the measured radiation dose rate D(107). In this equation, D(t) = D(107)·exp(0.693·(107-t)/110), 107 minutes is the time of measurement and 110 minutes is the halftime of ^18^F. The cumulative dose for an one hour operation, defined as C(1 h), is then C(1 h) ≅ (D(t_start_) + D(t_start_+1 h))/2, where t_start _is the starting time of the operation and in this study is equal to either 30 minutes or 3 hours. Similarly, the cumulative dose for a two hour operation, defined as C(2 h), is then C(2 h) ≅ C (1 h) + (D(t_start_+1 h) + D(t_start_+2 h))/2. This same method can be applied to the cumulative dose for any operation lasting more than 2 hours, defined as C(n). In any such situation, C(n) ≅ C (n-1) + (D(t_start_+n-1) + D(t_start_+n))/2, with n defining the duration of the operation.

## Results

Preoperative CEA was elevated in 11 patients (mean 28.8 ng/mL, ranging from 5.2 to 181.1 ng/mL). In 8 patients, preoperative CEA levels were within normal range. Preoperative CEA levels were not available in two patients. The normal range for CEA in our clinical laboratory is 0 to 5.0 ng/mL (chemiluminescent immunoassay).

**Figure 1:**
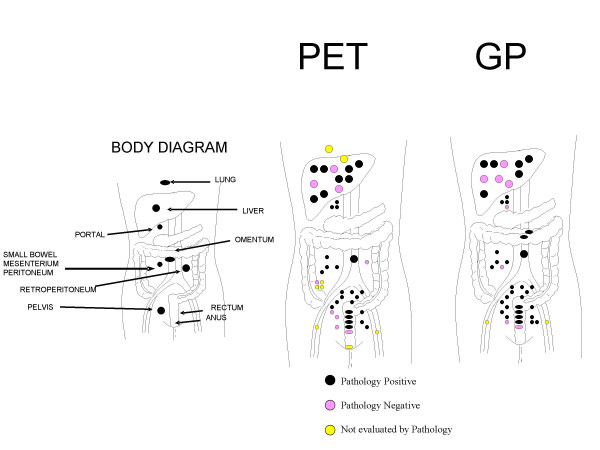
Location of the lesions detected by PET and GP.

**Figure 2:**
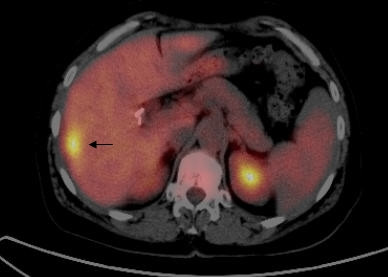
PET/CT fusion image on transaxial section demonstrates a hypermetabolic lesion (SUV_max _: 8.2, Patient number 14) in the segment 6 of the liver (arrow) which could not be detected by GP. Final pathology was consistent with CRC metastasis.

Preoperative FDG-PET and intraoperative GP detected 48 lesions (21 patients) and 45 lesions (17 patients), respectively. Intraoperative GP was negative for GP activity in four patients. A total of 50 presumed sites of recurrent disease from 20 patients were resected and sent for histopathologic evaluation. Six sites of presumed recurrent disease identified by preoperative FDG-PET, but which were not accessible or were negative by intraoperative GP, were not resected. Likewise, two sites of presumed recurrent disease identified in the external inguinal region by both preoperative FDG-PET and intraoperative GP were not resected. Thirty-seven of the 50 tissues specimens (from a total of 17 patients) were histological-proven tumor positive and 13 specimens (from a total of 8 patients) were histological-proven tumor negative (Table 2). When correlated with final pathology, the number of the true positive lesions by preoperative FDG-PET and intraoperative GP were 31 and 35, respectively (Table 3). The number of false positive by preoperative FDG-PET and intraoperative GP were nine and eight, respectively (Table 3). Both preoperative FDG-PET and intraoperative GP accurately detected 29 of 37 pathologically proven sites of recurrent tumor. The location of the lesions by preoperative FDG-PET and intraoperative GP are shown on body diagrams in Figure 1. Intraoperative GP detected six additional tumor foci that were not identified on preoperative FDG-PET. These tumor foci were all less than one centimeter in size and were generally located within the omentum and deep pelvis. Preoperative FDG-PET scan demonstrated two additional liver metastases in two patients which were not detected by intraoperative GP (Figure 2).

**Table 2:** Location and pathology findings of the lesions detected by PET and GP

**Pt No**	**PET [Location(No)]**	**GP [Location(No)]**	**Pathology**	**Follow-up**
1	Liver (3)	Liver (3)	+++ PET&GP	
	Portal (3)	Portal (3)	+++ PET&GP	
	Rectum	Rectum	+ PET&GP	
2	Liver	Liver	+ PET&GP	
3	Rectum	Rectum	+ PET&GP	
4	Pelvis	-	- PET	- CEA
5	Rectum	Rectum	+ PET&GP	
	Pelvis	-	- PET	
6	Rectum	Rectum	+ PET&GP	
7	Liver	-	No specimen sent	
	RLQ	-	- PET	
	Pelvis	Pelvis (3)	+ PET; +++ GP	
8	RLQ (2)	-	No specimen sent	
	Pelvis	Pelvis	+ PET&GP	
	-	Liver (2)	-- GP	
9	Pelvis	Pelvis	+ PET&GP TV adenoma in colon	
10	Small bowel	Small bowel	+ PET&GP	
	Pelvis	Pelvis (3)	+ PET; +++ GP	
	-	Omentum	+ GP	
11	Lung	Not accessible	No specimen sent	+ PET, + CEA, + Path.
12	Pelvis	Pelvis	+ PET&GP	
13	Liver (3)	Liver (2)	++- PET; +- GP	
	-	Small bowel	- GP	
14	Liver	-	+ PET	
15	Liver	Liver	- PET&GP	- CEA, - PET, - Path
	Retoperitoneum	-	- PET	
	Pelvis	Pelvis	- PET&GP	
	RLQ	-	No specimen sent	
	-	Portal	- GP	
16	Pelvis	Pelvis	+ PET&GP	
17	Pelvis	Pelvis	+ PET&GP	
18	Liver	-	- PET	+ CEA
	Anus	-	No specimen sent	
19	Peritoneum	Peritoneum	+ PET&GP	
	-	Omentum	+ GP	
20	Rectum	Rectum	- PET&GP	
	Pelvis (2)	Pelvis (2)	++ PET&GP	
	Liver	Liver	+ PET&GP	
21	Abdominal wall	Abdominal wall	+ PET&GP	
	Pelvis (2)	Pelvis (2)	++ PET&GP	
	Mesenterium	Mesenterium	+ PET&GP	
	Peritoneum	Peritoneum	+ PET&GP	
	Inguinal (2)	Inguinal (2)	No specimen sent	

Pt No (Patient number); RLQ (Right lower quadrant); TV (Tubulovillous); Path (Pathology); - (Negative); + (Positive).

**Table 3:** Number of the lesions detected by PET and GP

	**TP**	**FP**	**NE**
**PET**	31	9	8
**GP**	35	8	2
**Both PET and GP Positive**	29	4	2
**Only PET Positive but GP Negative**	2	5	6
**Only GP Positive but PET Negative**	6	4	0

NE: Not evaluated by pathology

In three patients (patient number 4, 15, and 18), histopathologic evaluation failed to demonstrate recurrent tumor within any of the tissues resected. In patient number 4, the preoperative CEA level was 1.6 ng/mL and the preoperative FDG-PET scan demonstrated a hypermetabolic focus in the pelvis. However, histopathologic evaluation of the resected tissues failed to demonstrate recurrent tumor. This patient's multiple postoperative CEA levels during 16 months of follow-up have remained within normal limits. In patient number 15, the preoperative FDG-PET scan demonstrated multiple hypermetabolic foci within the liver, retroperitoneum, and pelvis. The preoperative CEA level was only 0.5 ng/mL. Intraoperative GP was positive for the liver, pelvis, and portal region. In this patient, histopathologic evaluation of all resected tissues failed to demonstrate recurrent tumor. This patient's follow-up CEA level at 2 months remained within normal limits. A subsequent follow-up FDG-PET scan in 6 months demonstrated a decrease in metabolic activity in these lesions and additional histopathology following subsequent surgical resection of lesions within the liver and lymph nodes was also negative. In patient number 18, the preoperative CEA level was 9.5 ng/mL and the preoperative FDG-PET scan demonstrated suspicious lesions in the liver and anal region. However, intraoperative GP was negative and tissue sampling from the liver was negative for tumor recurrence. In this case, the CEA level at a time two months follow-up was noted to have further increased.

In patient number 11, the preoperative CEA level was 9.2 ng/mL and the preoperative FDG-PET scan demonstrated only a hypermetabolic right lung lesion. This was not accessible with the intraoperative GP and was not resected. Likewise, intraoperative GP was also negative within the abdomen and pelvis. Therefore, no specimens were sent on patient number 11. A follow-up FDG-PET scan demonstrated progression of the right lung lesion and a further increase in the CEA level. This lesion was subsequently surgically resected and histopathology confirmed that this was a CRC metastasis.

In three patients in which the histopathology of the tumor recurrence was specifically classified as mucinous adenocaricoma, both preoperative FDG-PET and intraoperative GP were able to detect all sites of tumor recurrence. In two of these patients, the SUV_max _and GP counts were markedly elevated and in one patient there was only a mild increase in the SUV_max _and GP counts.

The mean LC/BC ratios in true positive and false positive lesions by intraoperative GP were 1.68 ± 0.57 and 2.36 ± 2.50, respectively (P = 0.50071). The mean SUV_max _in true positive and false positive lesions were 8.27 ± 4.76 and 3.65 ± 1.22, respectively (P = 0.00021).

The estimated radiation doses to the surgeon during GP surgery are shown in Tables 4 and 5. A maximum radiation dose of approximately 606 μGy would occur to a surgeon who maintains a distance of 0.152 m away from the patient during a five hour operation that started 30 minutes after ^18^F FDG administration (Table 4), while a minimum radiation dose of less than 10 μGy would occur to a surgeon who maintains a distance of 0.914 m away from the patient during an one hour operation that started three hours after ^18^F FDG administration (Table 5). All other proposed operation scenarios would fall somewhere between these two extremes.

**Table 4:** Cumulative radiation dose in GP surgeries starting approximately 30 minutes after ^18^F FDG administration

	Cumulated Dose (μGy) @ 0.152 m	Cumulated Dose (μGy) @ 0.305 m	Cumulated Dose (μGy) @ 0.914 m
1 hr	224.5	144.0	25.4
2 hr	378.4	242.7	42.8
3 hr	483.8	310.3	54.7
4 hr	556.0	356.6	62.8
5 hr	605.5	388.4	68.4

**Table 5:** Cumulative radiation dose in GP surgeries starting approximately 3 hours after ^18^F FDG administration

	Cumulated Dose (μGy) @ 0.152 m	Cumulated Dose (μGy) @ 0.305 m	Cumulated Dose (μGy) @ 0.914 m
1 hr	87.3	56.0	9.9
2 hr	147.0	94.3	16.6
3 hr	188.0	120.6	21.2
4 hr	216.1	138.6	24.4
5 hr	235.3	150.9	26.6

## Discussion

The accurate assessment of the location and extent of all sites of intraabdominal and intrapelvic tumor recurrence, as well as of all sites of distant metastatic disease, plays a critical role in optimizing therapeutic strategies for delaying the development of further disease in CRC patients. Utilizing such an approach can lead to improvement in long-term outcome for CRC patients faced with this far too common sequela.

Numerous studies have demonstrated that FDG-PET has high sensitivity and high specificity for the detection of tumor recurrence in CRC patients with rising CEA levels in which there are no identifiable sites of tumor recurrence by standard anatomical imaging methods [11-14]. Particularly, combined PET/CT is superior to CT and MRI for the detection of CRC local recurrences, as well as metastatic disease to the liver and other distant sites [15-21]. However, the current FDG-PET scanning systems have several limitations. One of the most important limitations of FDG-PET scanning is its low sensitivity in detecting small sized lesions (23). FDG-PET has limited ability to detect lesions measuring less than 5 to 10 mm in size. A second limitation of FDG-PET imaging is the limited ability to assess local tumor invasion into the surrounding tissues. Strictly speaking, PET only imaging cannot identify local tumor invasion into the surrounding tissues secondary to the absence of anatomical correlation, as can be provided by combined PET/CT imaging. Nevertheless, while combined PET/CT imaging may be helpful in this regard if deeper tumor invasion is present, it will not be able to assess more limited tumor invasion. An additional limitation of FDG-PET imaging is its limited sensitivity for the detection of tumors that display a low metabolic activity [17]. Finally, ^18^F FDG is limited by the fact that it is not cancer-specific, and resultant physiological uptake into benign tissue processes, such as infection and inflammation, can result in the identification of areas of increased uptake of ^18^F FDG.

Over the last 20 years, intraoperative GP detection of tumor has been extensively studied in CRC patients by Martin and his colleagues at The Ohio State University [24-29,31-42]. For most of these studies, a mechanism of cancer-specific detection was designed in which monoclonal antibodies against TAG-72 tumor-associated glycoprotein that were coupled with iodine-125. This technology has been trademarked as Radioimmunoguided Surgery (RIGS). RIGS has been shown to be very useful in determining the surgical resection margins, in defining the extent of nodal involvement, and as a method of immediate intraoperative staging [24,26,27]. All these aspects of RIGS ultimately lead to a reduction in the risk for future recurrences. The use of RIGS was also been shown to impact upon the intraoperative strategy, thus altering the type of surgical procedure performed in some cases and avoiding major extirpation procedures in other cases in which chemotherapy and radiotherapy were decided to be given [26,27]. Finally, RIGS has been instrumental in defining sites of occult disease [24-27], in which the RIGS positive tissues have been shown to contain micrometastatic disease by immunohistochemistry despite the fact that these same tissues were negative on routine hematoxylin and eosin (H&E) staining (33).

In more recent years, several groups have utilized ^18^F FDG as a tumor targeting agent for intraoperative GP studies. In patients with CRC, intraoperative GP results have been shown to correlate well with preoperative FDG-PET results [28-30]. The combination of these two techniques has helped to improve the surgeon's ability to obtain a complete resection of primary or residual tumors [28]. The authors of these studies have emphasized the point that intraoperative GP removal of all ^18^F FDG positive tissue ensures for more complete removal of the tumor burden as compared to the surgeons' more traditional hands-on approach of assessing and resecting presumed sites of tumor. In this regard, Essner et al used ^18^F FDG and the intraoperative GP to assist in differentiating normal tissue from tumor-bearing tissue in patients with metastatic CRC and melanoma [30].

In our current study, intraoperative GP tumor localization correlated well with the findings on preoperative FDG-PET imaging. Over three-fourths (29 of 37) of the histopathologic confirmed sites of tumor recurrence were detected by both preoperative FDG-PET imaging and intraoperative GP. The intraoperative GP detected more sites of recurrent tumor within the abdomen and pelvis. These tumors were less than one centimeter in size and were located within the omentum and deep pelvis. In this particular regard, the main advantage of the intraoperative GP over that of preoperative FDG-PET imaging is the ability to have the intraoperative GP in close proximity to the suspected site of recurrent disease at the time of surgery. This same contention has also been put forth by Barber et al, in which they previously showed that a sodium iodide based scintillation probe was more sensitive in detecting small, deep tumors than was a gamma camera over a wide range of conditions, provided that the probe was placed within one centimeter of the tumor (43).

In our current study, preoperative FDG-PET scan accurately demonstrated liver metastases in two patients with histopathology proven metastasis which could not be detected by the intraoperative GP. It is apparent that tumors located more deeply within the liver may not be optimally detected by intraoperative GP, given the fact that the physiologic activity of ^18^F FDG within the liver in areas overlying and surrounding the presumed tumor may interfere with its accurate intraoperative GP detection. Although physiologic activity of ^18^F FDG within the liver can also be problematic in FDG-PET imaging, the process of separating the overlying and underlying activity into sequential tomographic planes allows FDG-PET imaging to increase image contrast and improve lesion detection and localization within deeper tissue planes.

In our current study, preoperative FDG-PET demonstrated a lung metastasis in one patient. This pulmonary lesion was not accessible with the intraoperative GP. Nevertheless, previous studies have demonstrated that preoperative FDG-PET can be a valuable imaging tool to detect distant metastases and to help differentiate isolated resectable recurrence from that of disseminated metastatic disease and thus help select appropriate CRC patients who would benefit from attempted surgery resection [16,21].

A recognizable limitation to both FDG-PET imaging and intraoperative GP detection is the physiologic ^18^F FDG activity which can be demonstrated within nonmalignant tissues. This is especially evident in the scenario in which increased ^18^F FDG activity is seen in associated with infectious and/or inflammatory changes within nontumor-bearing tissues. Such a scenario creates false positive results with both preoperative FDG-PET imaging and intraoperative GP detection techniques [44]. Likewise, it is well established that mucinous tumors are more difficult to identify with FDG-PET imaging, likely secondary to a lower metabolic activity of such tumors. It has been previously reported that the sensitivity of FDG-PET imaging for the detection of mucinous carcinoma is significantly lower than that of nonmucinous carcinoma (58% and 92%, respectively) [17]. This would also be the expectation for intraoperative GP detection of ^18^F FDG. Nevertheless, in our current study, the sites of tumor recurrence were accurately detected by both FDG-PET imaging and intraoperative GP detection in those three patients whose CRC recurrence represented mucinous adenocarcinoma. Although previous studies suggest that both preoperative FDG-PET imaging and intraoperative GP detection may not as effectively identify mucinous tumors, the surgeon's ability to position the intraoperative GP in close proximity to sites of suspected tumor recurrence may ultimately make intraoperative GP detection more efficient for the detection of mucinous tumors as compared to preoperative FDG-PET imaging.

The estimated radiation dose received by a surgeon during a single ^18^F FDG GP surgery was much lower than the occupational limit of 50 mGy/yr (50,000 μGy/yr) and even below the limit for the general public of 1 mGy/yr (1,000 μGy/yr), as determined and mandated by Federal regulations. With no special shielding from radiation, the radiation dose should not cause health hazard to any given surgeons during a single ^18^F FDG GP surgery. However, for surgeons who perform multiple ^18^F FDG GP operations, the cumulative radiation dose will be the sum of each single radiation dose. Depending on how many ^18^F FDG GP operations are performed by a given surgeon during a finite period of time, the cumulative radiation dose to the surgeon may significantly increase and may even exceed regulatory limits. In order to comply with Federal regulations, the number of ^18^F FDG GP operations performed by a given surgeon during a finite period of time will need to be monitored and limited in such a fashion as to keep the cumulative radiation dose to the surgeon to within the occupational limit of 50 mGy/yr (50,000 μGy/yr).

## Conclusion

The combined use of preoperative FDG-PET imaging and intraoperative GP detection is potentially helpful to the surgeon as a roadmap for accurately locating and determining the extent of tumor recurrence in patients with CRC. While the intraoperative GP appears to be more sensitive in detecting the extent of abdominal and pelvic recurrence, preoperative FDG-PET imaging appears to be more sensitive in the detecting liver metastases. FDG-PET imaging also detects unexpected distant metastases.

## Competing interests

The author(s) declare that they have no competing interests.

## Authors' contributions

**IS **organized, wrote, and revised the manuscript. **EWM **was the supervising senior physician for the entire project. He designed the current study and collected the data. **EWM, SPP**,**OHA**,**EK**, **MB**, **SM**, **ZC**, **DAM**, **JZ**, **NCH**, and **MVK **assisted in the writing and editing of all aspects of this manuscript. All the authors have approved the final version of this manuscript.
